# Landau-Kleffner Syndrome, Attention-Deficit/Hyperactivity Disorder (ADHD), and Viral/Autoimmune Encephalitis: Challenges in the Diagnosis and Management of a Six-Year-Old Boy

**DOI:** 10.7759/cureus.52133

**Published:** 2024-01-11

**Authors:** Lujain Althagafi, Rahaf Al Fuhayd, Fatimah K Almeathem, Razan A Almeshal, Lma J Al-Amri, Wessal A Mustafa

**Affiliations:** 1 Psychiatry and Behavioral Sciences, King Abdullah bin Abdulaziz University Hospital (KAAUH), Riyadh, SAU

**Keywords:** autoimmune encephalitis, psychiatry, asd, adhd, pediatric neurology, landau-kleffner syndrome

## Abstract

In this case, we discuss the difficulties and challenges faced when diagnosing and treating a six-year-old boy presenting with abnormal behaviors and difficulty in concentration and inattentiveness, followed by regression of expressive language. These symptoms were then followed by hyperactivity, bouts of anger, and difficulty sleeping. The patient was seen by a psychiatrist, and he was diagnosed with attention-deficit/hyperactivity disorder (ADHD) initially and treated with little to no improvement. He was then recommended to see a neurologist by his psychiatrist and underwent a series of investigations, which included the following: brain MRI with contrast, magnetic resonance (MR) spectroscopy, cerebrospinal fluid (CSF) routine, CSF N-methyl-D-aspartate (NMDA) receptor antibody and glutamate decarboxylase (GAD) antibody analysis, and a CSF meningitis multiplex polymerase chain reaction, thyroid function, ammonia, lactate, creatine kinase, liver function, and metabolic screening of urine organic acids (UOAs), all of which revealed no abnormalities. Vitamin D was low at 38 ng/ml (>50). An electroencephalogram (EEG) done under standard conditions and provocative stimulation was abnormal with bilateral central and frontal discharge and more activity on the right side, revealing a background activity of moderately organized alpha waves (8-13 Hz) and bursts of sharp and slow wave activities that were accentuated by photic stimulation. Polysomnography showed poor sleep efficiency of 84.7%, and rapid eye movement (REM) stage was not reached due to interrupted sleep. He was then diagnosed with epileptic encephalopathy and Landau-Kleffner syndrome (LKS). The patient was prescribed sodium valproate, intravenous immunoglobulin (IVIG), and pulse steroids, with no major improvement, risperidone was added but was poorly tolerated, and the dose tapered off and eventually discontinued. Methylphenidate was the prescribed starting at 5 mg, and the dose was gradually increased to 20 mg/day given separately as 10 mg twice a day. A week later, melatonin 2 mg was added. Three months, later the EEG was repeated and was normal, and sodium valproate was tapered off and eventually discontinued. Later on that year, he was diagnosed with COVID-19 and developed acute myositis as a complication, methylphenidate was stopped, and only sertraline and melatonin were continued. MRI was repeated only this time, showing evidence of viral/autoimmune encephalitis (AIE) sequela, and IV corticosteroids were given alongside IVIG. He was discharged on prednisolone, and a month later, major improvements were seen in all aspects.

## Introduction

Landau-Kleffner syndrome (LKS) is a rare acquired neurological pediatric disorder that is characterized by acquired epileptic aphasia in those with age-appropriate speech development [1]. Typical manifestation of LKS occurs in children aged three to seven years old, with a progression over days or weeks [2]. The earliest discovery of a “syndrome of acquired aphasia with convulsive disorder” was in 1957, with a total of six cases in children reported by Landau and Kleffner [1]. In an editorial article published in the 1990s, Landau conveyed the hope that modern medicine would eventually reach a universally accepted diagnosis criteria, etiology, pathophysiology, and rational treatment for LKS [3].

In the latest version of International Classification of Diseases 10th Revision (ICD-10), LKS is listed as a disorder in which the child, having previously made normal age-appropriate development in language and speech, loses both receptive and expressive language skills but retains general intelligence [2]. This type of acquired childhood aphasia is incredibly rare; in Japan, there is one case per million children [4]. Unfortunately, the available literature does not provide enough information about incidence and prevalence worldwide [5]. All children with LKS have abnormal electroencephalograms (EEGs) that are consistent with a diagnosis of epilepsy, although only 70% of them experience clinical seizures [6]. More than half of patients are left with a permanent receptive language deficit, while a few may regain language abilities [2].

Autoimmune encephalitis (AIE) is an autoimmune disease marked by an inflammation process in the brain, which may be caused by various mechanisms, including an infective organism or autoimmune reactions [7]. Normally, pediatric patients diagnosed with AIE are usually medically free and present with a sudden rapid onset of neurological and psychiatric symptoms and prodromal symptoms, including high temperature, which occur in more than half of these patients [8-11]. Abnormal movements may also present initially along with seizures, both of which are among the most frequent symptoms [9]. The diagnosis should be suspected in kids who present with acute local or generalized neurological deficits, cognitive impairment, developmental difficulties, and psychiatric issues with or without seizures. These kids must be investigated extensively. This diagnosis should not be made based only on serology testing, as some may be negative [9].

A number of studies have shown how effective corticosteroids and intravenous immunoglobulin (IVIG) are as first-line medications in the management of AE. Plasma exchange may also count as first-line management, although most commonly, in pediatric patients, it is kept for patients with severe symptoms, as those who need to be urgently admitted to ICU [12].

## Case presentation

The patient was a six-year-old boy, previously healthy, with an unremarkable perinatal history. The patient’s parents noticed difficulty in sleep and hyperactivity starting along with poor concentration and inattentiveness at the age of six years and eight months (July 2021), alongside hyperactivity when sitting on a chair, and difficulty falling asleep, which resulted in unorganized sleep time but normal sleep hours. These symptoms were followed by a change in personality, isolation, loss of eye contact, no response to commands, interrupted speech, incapability of dressing himself, lost appetite, having conversations back and forth with himself, and specific movements of his eyes that were noted during August 2021. Later on that month, there were new symptoms, which include bouts of anger toward his mother, low mood, loss of interest, and lack of reactions or expressions. The patient had no history of headaches or seizures, and the systemic review was unremarkable. His past medical history was also unremarkable. The patient’s parents were consanguineous, but there was no family history of autism, attention-deficit/hyperactivity disorder (ADHD), genetic disorders, neurological diseases (including seizures), or autoimmune diseases.

The initial visit was in September 2021 with a psychiatrist who diagnosed the patient with ADHD clinically and prescribed him sertraline 50 mg and atomoxetine 10 mg. No improvement was reported, and the patient's symptoms were worsening. He went back to his psychiatrist and was recommended to visit a neurologist for further evaluation.

In November 2021, the patient was evaluated at a neurology clinic in Riyadh, Saudi Arabia. This examination recorded that the patient was conscious, alert, oriented, hyperactive, and nonverbal. It revealed that the patient had impaired comprehension and a short attention span. The cranial nerves and motor examinations were normal. His reflexes were +2 in all limbs, and there was a downward Babinski response. Cerebellar signs were negative, there were no neurocutaneous stigmata, and his gait was normal. He was admitted for further investigations. The following investigations were carried out: brain MRI with contrast, magnetic resonance (MR) spectroscopy, cerebrospinal fluid (CSF) routine, CSF N-methyl-D-aspartate (NMDA) receptor antibody, glutamate decarboxylase (GAD) antibody analysis, and a CSF meningitis multiplex polymerase chain reaction. These tests revealed no abnormalities, and no other CSF tests were done to rule out other AIE. The following laboratory investigations were conducted: thyroid function, ammonia, lactate, creatine kinase, and liver function, all of which revealed no abnormalities. The metabolic screen (urine) of urine organic acids (UOAs) was normal. Vitamin D was low at 38 ng/ml (>50).

An electroencephalogram (EEG) was done under standard conditions and provocative stimulation, revealing a background activity of moderately organized alpha waves (8-13 Hz) and bursts of sharp and slow wave activity that were accentuated by photic stimulation. It was concluded that the EEG was abnormal during both wakefulness and sleep, with bilateral parito-centro-temporal discharge and more activity on the right side that seen during sleep frequent but not continuous. Based on the EEG results, the diagnosis of LKS was given, and sodium valproate (Depakine) 110 mg orally twice a day for five months along with IVIG 40 gm for two days and IV corticosteroids (methylprednisolone) 400 mg for three days was initiated. Improvement was reported in his mood, he had less angry bouts and was more attentive only for a week, and then he was back to his baseline. Polysomnography was done, and the results included the following: poor sleep efficiency of 84.7%, and the patient did not reach rapid eye movement (REM) stage due to interrupted sleep; however, there was a normal nocturnal gas exchange.

He was referred to the child development center for psychological and psychiatric assessment, along with a speech pathology assessment to screen for metabolic disorders. The differential diagnoses were childhood disintegrative disorder, autism spectrum disorder (ASD), and ADHD. The psychiatrist diagnosed the patient with ADHD, atomoxetine was discontinued, and the patient was scheduled for behavioral therapy. Methylphenidate was added at the dose of 5 mg once a day for the first week and then 5 mg twice a day for the second week. Then, 10 mg was given at the morning and 5 mg at evening for the third week and eventually 10 mg twice a day for the fourth week. Thereafter, melatonin was also added at the dose of 2 mg once daily. After one week, improvement was seen in regard to attentiveness, loss of interest, and lack of sleep, but he remained aggressive and isolated and makes little to no eye contacts with siblings and/or parents.

A month later, risperidone was added starting with a dose of 0.25 mg once daily for a week, then 0.5 mg once daily for a week, and then 0.75 mg once daily for a week. It was reported after that he was developing side effects from risperidone, such as dizziness, dry mouth, and restlessness, and the dose was decreased to 0.5 mg once a day. A week later, a repeat EEG was obtained and was normal, and risperidone was decreased to 0.25 mg once a day and eventually discontinued due to side effects.

By the end of March 2022, he was still on sodium valproate, methylphenidate, sertraline, and melatonin, and a repeat EEG was done and was normal. Sodium valproate was gradually tapered off and stopped by then end of April 2022. The rest of the medications he was on were continued, and a repeat EEG done in the mid of May 2022 was normal. By the beginning of June, he was diagnosed with COVID-19 and developed acute myositis as a complication, his creatine kinase (CK) was 8742 U/L, and was admitted to PICU for three days and then transferred to the pediatric ward in King Abdullah Bin Abdulaziz University Hospital in Riyadh, Saudi Arabia. Soon after, methylphenidate was gradually decreased and eventually discontinued.

In May 2022, the patient was seen in the a child psychometric clinic and was classified as having a severe delay in his social-emotional development. He was scheduled to receive family-based behavioral interventions and social-emotional interventions.

**Figure 1:**
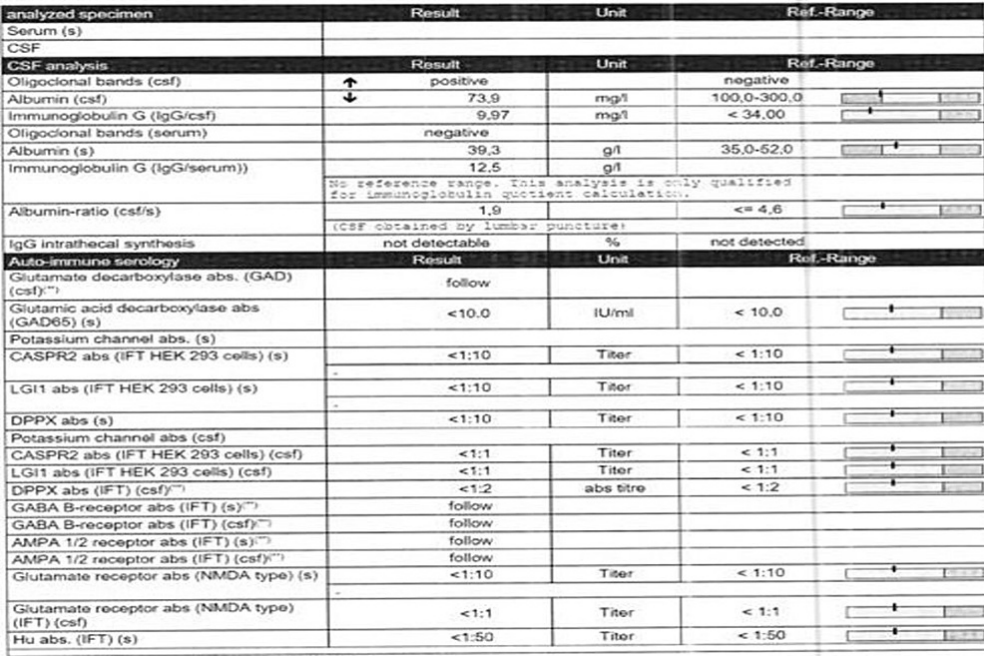
Cerebrospinal fluid (CSF) analysis results

**Figure 2:**
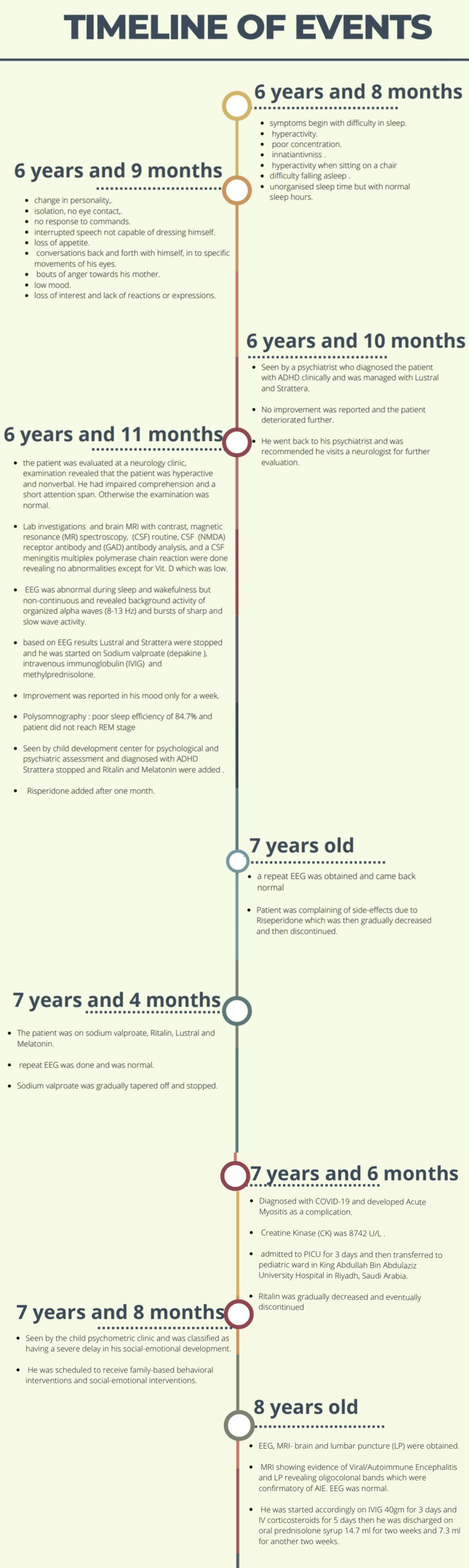
Timeline of events

In mid December 2022, the following investigations were done: EEG, MRI-brain and lumbar puncture, and EEG. The MRI showed evidence of viral/AIE, which included increased bilateral anterior temporal, hippocampal, and insular fluid-attenuated inversion recovery (FLAIR) signal intensity with no associated volume loss or enhancement. These findings could suggest sequela of previously treated viral/AIE that could have been missed in the previous MRI. LP done revealed oligocolonal bands, which were confirmatory of AIE (see Figure 1). EEG was normal. He was started accordingly on IVIG 40 gm for three days and IV corticosteroids for five days, and then he was discharged on oral prednisolone syrup 14.7 ml for two weeks and 7.3 ml for another two weeks (see Figure 2). After a month during follow-up, the patient’s family reported major improvements in all aspects.

## Discussion

LKS is challenging to diagnose as it may be difficult to differentiate from ASD, pervasive developmental disorder, intellectual disability, ADHD, childhood schizophrenia, and behavioral problems [13]. Symptoms of these conditions may overlap or co-exist in some patients and cause a delay in diagnosis, as they did in this case.

LKS is significantly associated with epileptiform EEG abnormalities. The EEG manifestations seen in LKS are not related to a single rhythmic waveform but are commonly associated with extended durations of wave discharges and are frequently stimulated during sleep. Generalized, focal, bilateral, multifocal spike, or spike-wave discharges have all been observed in patients with LKS. These abnormalities frequently appear to be bilateral or lateralized more to the right hemisphere [14]. The EEG conducted on this patient revealed findings that correlated with LKS, and these EEG manifestations helped diagnose the patient with LKS after excluding other causes, such as AIE. A significant number of individuals with LKS experience evident seizures during the course of syndrome development, whereas 20-30% do not develop evident seizures or behavioral manifestations of seizures [13]. In this case report, there was no history of seizures noted in the patient.

A number of children with LKS show symptoms of decreased attention and/or hyperactivity. These symptoms range from mild to severe, affecting the patient’s daily activities and meeting the diagnostic criteria for ADHD [15]. In this patient, the symptoms of abnormal behavior, difficulty in concentration, and inattention were significant and met the criteria for a diagnosis of ADHD. The diagnosis was made and treatment with methylphenidate, melatonin, and sertraline was given, which could explain the partial improvement reported by our patient.

Medications that have been reported to be beneficial in the management of LKS mainly include empirical treatment with valproate sodium or diazepam. Other antiepileptic drugs, such as ethosuximide, IVIG, and corticosteroids (either orally or high intravenous pulse doses), may be used. It should be noted that corticosteroid therapy should be initiated within one to two months of the initial diagnosis of LKS. Furthermore, only a small number of patients will experience an initial improvement with IVIG. In addition to medication, speech therapy and behavioral interventions should be provided in all patients to manage LKS [13]. In this case report, the patient received initial empirical treatment with no significant improvements being noticed. Improvements began to be observed following the initiation of behavioral therapy and managing the ADHD with methylphenidate.

In AIE, pediatric patients are usually medically free and develop initially acute or subacute symptoms of neurological and psychiatric symptoms [16] like our patient.

Diagnosing AIE can be a challenge because many of the clinical symptoms may overlap whether with other types of AIE, brain diseases, psychiatric disorders, metabolic diseases, and infections. In pediatrics, it is considered even more difficult due to constant, complex behavioral changes occurring in childhood, let alone the inability of these patients to accurately describe their symptoms [16]. According to the International Encephalitis Consortium 2013, the diagnostic criteria for encephalitis of suspected autoimmune or infectious pathophysiology demand a patient to have an altered behavior that has lasted more than 24 hours with other causes excluded. To confirm the diagnosis, there should be at least three minor criteria, including high temperature within three days before the patient’s presentation, new development of local neurological symptoms/signs, increased CSF white blood cell count, and acute new findings indicative of encephalitis in neurological imaging methods. EEG abnormalities are consistent with those of encephalitis [17]. In our patient, none of these were met initially, and hence the diagnosis was delayed and so was the treatment.

## Conclusions

This case demonstrates the challenges of diagnosing a child showing signs and symptoms of LKS, ADHD, ASD, and AIE. The diagnosis of LKS was given initially based on the characteristic findings in the EEG, and treatment with sodium valproate, IVIG, and corticosteroids was started, which did not result in a partial resolution of symptoms until symptoms continued to appear. Based on the patient’s symptoms of abnormal behavior, difficulty in concentration, and inattention, as well as global delay in social-emotional development and intellectual delay, the criteria for both ADHD and ASD were met, the diagnosis was therefore given, and the treatment started accordingly. However, the patient’s symptoms were not completely resolved, which could be explained by the missed findings of AIE in the first MRI.
